# Capsaicin attenuates the effect of inflammatory cytokines in a HaCaT cell model for basal keratinocytes

**DOI:** 10.3389/fphar.2024.1474898

**Published:** 2024-10-14

**Authors:** Maria Fernanda Cervantes Recalde, Jana Schmidt, Cristina Girardi, Marco Massironi, Markus Leo Rechl, Joachim Hans, Dominik Stuhlmann, Veronika Somoza, Barbara Lieder

**Affiliations:** ^1^ Institute of Physiological Chemistry, Faculty of Chemistry, University of Vienna, Vienna, Austria; ^2^ Vienna Doctoral School in Chemistry (DoSChem), University of Vienna, Vienna, Austria; ^3^ Symrise Srl, CUTECH, Padova, Italy; ^4^ Christian Doppler Laboratory for Taste Research, Faculty of Chemistry, University of Vienna, Vienna, Austria; ^5^ Symrise AG, Holzminden, Germany; ^6^ Leibniz Institute of Food Systems Biology, Technical University of Munich, Freising, Germany; ^7^ Institute of Clinical Nutrition, University of Hohenheim, Stuttgart, Germany

**Keywords:** skin, inflammation, TRPV1, capsaicin, permeability

## Abstract

**Introduction:**

The resolution of the skin’s inflammatory response is only possible if its barrier function is restored. TRPV1 channel activation plays an important role during inflammation but the effect of this activation on the skin barrier under inflammatory conditions has not been clarified. We hypothesize that it could potentially aid the keratinocyte barrier by reducing inflammatory cytokine release and promoting tight junction development.

**Methods:**

To explore the role of TRPV1 activation in inflammation, we designed and optimized an *in vitro* model of keratinocytes with basal epidermal layer characteristics using HaCaT cells and TNFα to induce inflammation.

**Results:**

TNFα increased the gene expression of tight junction protein claudin 1 (CLDN1) by at least 2.60 ± 0.16-fold, in a concentration-dependent manner, over a 48 h period. The administration of a capsaicin pre-treatment reduced the *CLDN1* expression to 1.51 ± 0.16-fold during the first 6 h after TNFα induction, whereas IL-8 cytokine release was reduced 0.64 ± 0.17-fold. After 48 h, CLDN1 protein levels increased by a factor of 6.57 ± 1.39 compared to cells only treated with TNFα.

**Discussion:**

These results suggest that activation of TRPV1 by capsaicin can potentiate the increase in *CLDN1* expression and CLDN1 protein synthesis induced by TNFα in cultured keratinocytes, while reducing the release of IL-8.

## 1 Introduction

The skin tissue is a complex arrangement of biological compartments forming distinctive layers with unique relevance in the maintenance of a protein-lipidic barrier that protects the organism against external damage (McGrath et al., 2010). The outer layer of the skin, the epidermis, prevents the body from losing hydration and protects against the penetration of allergens, chemicals, or microorganisms into the body (Archer et al., 2010). The keratinocytes, the most abundant cells in the epidermis, form a physical and biochemical barrier that has the ability to interact with the external environment to communicate any threats to the immune and nervous systems (McGrath et al., 2010; Archer et al., 2010). This barrier plays a pivotal role in skin immunity and its integrity needs to be guaranteed for the return to homeostasis after the inflammatory response (Beck et al., 2022). Thus, even though keratinocytes are not the main effectors in either the immune reaction or sensorial perception, they are indispensable for the “first line of defense” that the skin provides.

The layers of the epidermis consisting on living keratinocytes, i.e., the *stratum basale* (SB)*,* the *stratum spinosum* (SS) and the *stratum granulosum* (SG), form a physical barrier that culminates in an enucleated protective layer, the *stratum corneum* (SC) (McGrath et al., 2010). The barrier function of the skin can be attributed mostly to the SC and the tight junctions (TJ) between keratinocytes of the SG in the epidermis (Archer et al., 2010). TJ proteins are key players that regulate the passing of molecules and ions through the keratinocyte layers sealing paracellular permeation and restricting molecules from the intercellular space (Katja Bäsler et al., 2016). The integrity of the TJs is known to be impaired in inflammatory skin diseases (Bergmann et al., 2020; Gruber et al., 2015; Gschwandtner et al., 2013) and they are targeted by pathogenic bacteria (Ohnemus et al., 2008). For this reason, it is important to understand how alterations in epidermal permeability are related to inflammation and what role TJs play.

Inflammation is characterized by the initial recognition of adverse stimuli through toll-like receptors (TLRs) in tissue resident macrophages or mast cells and the consequential release of inflammatory mediators, such as tumor necrosis factor alpha (TNFα) (Medzhitov, 2008). Activation of the keratinocyte TNFα receptor 1 (TNFα-R1) by TNFα secreted from immune cells or directly by pathogenic bacteria promotes the release of inflammatory cytokines including interleukin-8 (IL-8), interleukin 1α (IL-1α), interleukin-6 (IL-6), intercellular adhesion molecule (ICAM-1), etc., further exacerbating the response (Cristina Albanesi et al., 2005; Steinhoff et al., 2010; Claßen et al., 2011). IL-8 is commonly employed in literature as a marker for inflammatory skin disease (Claßen et al., 2011; Qiu et al., 2023-11; Patel et al., 2018) and acts as a chemotaxis factor for neutrophil infiltration in the tissue which has a direct effect on TJ remodeling (Harada et al., 1994; Parkos, 2016). Additionally, TNFα is known to enhance the skin barrier by ceramide promotion in the SC (Hatano et al., 2005). Meanwhile, its stimulatory effect on the synthesis of the TJ protein claudin 1 (CLDN1) has been demonstrated in cancer cells (Iida et al., 2022; Shiozaki et al., 2012). Among the various TJ proteins, CLDN1 stands out as essential for maintaining the main function of all epithelia (Furuse et al., 2002). The role of TNFα in the regulation of CLDN1 and skin permeability is still not fully understood as the connection of TNFα to TJ proteins has not been addressed in skin.

To attenuate the effects of TNFα on the skin barrier, a wide range of pharmaceutical compounds have been studied. Among these compounds is capsaicin, a known agonist of the transient receptor potential vanilloid 1 (TRPV1) ion channel, responsible for the sensation of noxious heat of certain ligands (Islas and Emir, 2017). TRPV1 is expressed in keratinocytes where it promotes the release of inflammatory cytokines upon activation (Southall et al., 2003-01). Interestingly, capsaicin has been shown to regulate TJ permeability, producing a detrimental effect on the skin barrier functionality of healthy tissue (Cong et al., 2012), but a protective effect during inflammation (Zhao et al., 2021). The latter property of capsaicin has not been addressed yet. However, in order to investigate the effects of capsaicin on TJ proteins and permeability, it is important to consider the characteristics of the different epidermal layers. Most studies focus on the effects of capsaicin on skin barrier recovery after tape stripping (Yun et al., 2011), which is closely related with the functionality of keratinocyte TJs. Although important, these results focus on the role of the SC in barrier function. Nevertheless, it is known that the SC barrier can only be guaranteed if the living keratinocyte layers are able to fulfil their TJ forming function. In this study, we aimed to create an *in vitro* cell model suitable to mimic the lower keratinocyte layers of the epidermis. To do this, we selected HaCaT as a keratinocyte cell line, which maintains some key metabolic properties of the primary skin cells and has functional TRPV1 channels (Huang et al., 2017; Kwon et al., 2018). In culture, different keratinocyte morphologies related to their differentiation to corneocyte can be obtained using different calcium concentrations in the culture medium (Wilson, 2014). In our model, the use of culture medium with low calcium concentration (0.06 mM Ca^2+^) led to rounded cubical cells, which group together but do not have contact with each other. This morphology simulates the inner layers of the epidermis (i.e., SB or SS), where keratinocytes have not yet formed TJs (Wilson, 2014). Because of this, our keratinocyte model enables the study of how TNFα influences the expression of TJ proteins, such as CLDN1, claudin 2 (CLDN2) and occludin (OCLN), before TJs are formed in the SG layer, as well as how capsaicin can modulate this response. Furthermore, the effects of capsaicin have been studied on the same markers expressed in *ex vivo* skin cultures treated with TNFα.

## 2 Materials and methods

### 2.1 Chemicals

The chemicals used for the stimulation of the cells included TNFα, capsaicin and capsazepine, all commercially obtained at a purity of ≥95%. TNFα (abcam) was dissolved in double-distilled water and applied at 2.5, 5, 10, 20 and 40 ng/mL in the different experiments. Capsaicin and capsazepine (Sigma Aldrich) were dissolved in dimethyl sulfoxide (DMSO) (Roth) and applied separately or in combination at 10 µM and 1 µM, respectively. The final DMSO concentration in the treatment never exceeded 0.2%.

### 2.2 *Ex vivo* skin sampling and treatment

A total of 36 skin biopsies, each with an 8 mm diameter, were obtained from surgical leftover residues produced after surgical intervention and made available by the donor after signing an informed consent form. Fat and hypodermis were removed from the skin explant, and the biopsies were excised using a sample punch. Skin samples were cultured in Williams’ Medium E (Sigma Aldrich) at air-liquid interface under classical cell culture conditions (37°C, in a 5% CO_2_/95% air-humidified incubator). After an acclimation period of 24 h the medium was replaced and the biopsies topically treated with two different capsaicin concentrations, namely, 100 µM and 500 μM, respectively, in vehicle (DMSO/PBS 1:25). Vehicle alone was used as a negative control. The biopsies were then covered with a CoTran™ cotran membrane (3M) and incubated for 24 h at 37°C and 5% CO_2_. The membranes were then removed and the surface of the epidermal biopsies was dried by blotting the remaining liquid with a cotton swab. Systemic treatment with 40 ng/mL TNFα solution in medium or medium alone (negative control) was subsequently performed for a period of 48 h. After this incubation period, the medium was collected for cytokine analysis and permeability was assessed on the skin biopsies.

### 2.3 Rhodamine assay

For the assessment of skin permeability, a rhodamine B (RhB) permeability assay was performed. Skin samples were topically treated with a 0.03% RhB (Sigma Aldrich) solution, covered with a CoTran™ membrane and incubated for 2 h at 37°C and 5% CO_2_. The membrane was removed and the excess RhB was blotted using a cotton swab. The skin samples were then cryo-fixed, embedded in FSC 22 frozen section media (Leica) and cut in 7 µm slices using a Leica CM1850 cryostat (Leica). The slices were mounted in glass using mounting medium Fluoroshield with DAPI (Sigma Aldrich) and photographed with a Leica DMi8 Microscope equipped with a DFC7000T digital camera (Leica). The images were taken at a ×200 magnification using a Texas red and UV filter, for RhB and DAPI, respectively. For each condition tested, the epidermis of 12 digital images, deriving from 6 skin biopsies, was analyzed by evaluating the fluorescence through ImageJ application (NIH, USA). The obtained value has been normalized upon the dimension of the selected area.

### 2.4 IL-8 ELISA for *ex vivo* samples

For the analysis of IL-8 cytokine release in the *ex vivo* skin model culture media of skin biopsies was collected after the respective treatment (see 2.2). Each treatment was repeated in triplicates and tested in four technical replicates. The analysis was done by means of an ELISA assay (MAX Deluxe Set Human IL-8 ELISA, BioLegend) following the manufacturer’s instructions and measuring the absorbance at a wavelength of 450 nm using a Varioskan Lux plate reader (Thermo Fisher Scientific).

### 2.5 Cell culture

HaCaT keratinocyte cells (Cell Lines Service) maintained under high calcium concentration (1.8 mM Ca^2+^) were cultured in Dulbecco’s Modified Eagle Medium (DMEM) (Thermo Fisher Scientific) supplemented with 10% FBS (Thermo Fisher Scientific) and 1% Penicillin/Streptomycin (Sigma Aldrich). The cells under low calcium concentration (0.06 mM Ca^2+^) were cultured in keratinocyte growth medium 2 kit (PromoCell) containing basal medium and a supplement mix comprising 0.004 mL/mL bovine pituitary extract, 0.125 ng/mL epidermal growth factor, 5 μg/mL insulin, 0.33 μg/mL hydrocortisone, 0.39 μg/mL epinephrine, 10 µ/mL transferrin and 0.06 mM CaCl_2_. A penicillin/streptomycin mixture was added to a concentration of 1% (v/v). The cell cultures were kept at 37°C and 5% CO_2_ in a humidified and sterile incubator.

### 2.6 Inflammation induction and treatment

For characterization of the inflammatory reaction, 4 days after seeding, some preliminary experiments were performed on the HaCaT keratinocytes maintained under low calcium conditions. A time-course experiment was carried out treating the HaCaT keratinocytes with 20 ng/mL TNFα for 3, 6, 12, 24 and 48 h, respectively. The obtained results suggested to adopt a time-treatment of 48 h, which was implemented in a dose-response experiment performed by treating the cells with 2.5, 5, 10, 20 and 40 ng/mL TNFα, respectively. The final experimental treatment was set up treating the HaCaT keratinocytes under different calcium concentrations with 20 ng/mL TNFα for 48 h. Pre-treatment, when administered, consisted of 10 µM capsaicin, or capsaicin in combination with 1 µM capsazepine, for 24 h before the TNFα treatment based on previous studies (Zhao et al., 2021; Walker et al., 2017). This time, samples were taken only at 6 or 48 h. DMSO was used as a negative control for all experiments.

### 2.7 Cell viability assay

Negative effects on cell viability after treatment with the different TNFα concentrations, 10 µM capsaicin, or 1 µM capsazepine, were excluded using the 3-(4,5-dimethylthiazol-2-yl)-2,5-diphenyl tetrazolium bromide (MTT) assay as previously described (Lieder et al., 2020). Cells were seeded at a density of $3\times 10^{4}$ cells/cm^2^ and treated with the respective compounds for a period of 24 or 48 h. The medium was removed, replaced with a 1 mg/mL MTT solution (Roth) in medium and incubated for 15 min (min) at 37°C and 5% CO_2_. The formazan crystals were thereafter dissolved in DMSO and the absorbance measured at 570 nm against a reference wavelength of 650 nm using a Tecan Infinite M200 plate reader (Tecan, Switzerland).

### 2.8 RNA isolation and RT-qPCR

RNA isolation was performed using the Monarch Total RNA Miniprep Kit (New England Biotechnologies) according to the protocol provided by the manufacturer. The RNA concentration of the respective samples was quantified using a Tecan Infinite M200 plate reader and measuring the absorbance at 260 nm. The 260/230 and 260/280 ratios were determined as quality controls for the purity of the RNA. A total of 0.5 µg of the isolated and purified RNA was used as a template for the transcription to cDNA using the LunaScript RT SuperMix Kit (New England Biotechnologies) as described by the manufacturer. A thermal cycler C1000 Touch™ (BioRad) was used for the transcription. The cDNA was diluted 1:5 with RNase free water and amplified by means of real time PCR (RT-qPCR) using Luna Universal qPCR Master Mix (New England Biotechnologies) on a Step-One Plus Device (Applied Biosystems). The primer pairs (Sigma Aldrich) for *CXCL8, IL6, CLDN1*, *CLDN2*, *OCLN* and two reference genes, *GAPDH* and *HPRT1*, used in this study are shown in Table 1. Newly designed primers were validated by sequencing. The analysis of the RT-qPCR data was performed with LinRegPCR (version 2020.0) to estimate the mRNA concentration (N_0_ values) using the measured C_T_ values and the PCR efficiency. The geometric mean of the reference genes, *GAPDH* and *HPRT1*, was used for normalization.

**TABLE 1:** Primer pairs used for the characterization of the gene expression induced by TNFα treatment.

Gene	Primer (forward) (5′-3′)	Primer (reverse) (5′-3′)	Source
*CXCL8*	ACT​GAG​AGT​GAT​TGA​GAG​TGG AC	AAC CCT CTG CAC CCA GTT TTC	(Soares et al., 2014/10)
*IL6*	AAA​CAA​CCT​GAA​CCT​TCC​AAA​GA	GCA​AGT​CTC​CTC​ATT​GAA​TCC​A	(Walker et al., 2017)
*CLDN1*	CCA​GTC​AAT​GCC​AGG​TAC​GAA	CAC​ACG​TAG​TCT​TTC​CCG​CT	Designed with Primer Blast
*CLDN2*	CGG​GAC​TTC​TAC​TCA​CCA​CTG	GGA​TGA​TTC​CAG​CTA​TCA​GGG​A	Spandidos et al. (2010)
*OCLN*	GTC​TAG​GAC​GCA​GCA​GAT​TG	CTG​GCT​GAG​AGA​GCA​TTG​GT	Designed with Primer Blast
*GADPH*	AGG​TCG​GAG​TCA​ACG​GAT​TTG	GGG​GTC​ATT​GAT​GGC​AAC​AAT​A	(Walker et al., 2017)
*HPRT1*	CCT​GGC​GTC​GTG​ATT​AGT​GA	CGA​GCA​AGA​CGT​TCA​GTC​CT	Lieder et al. (2020)

### 2.9 IL-8 ELISA for *in vitro* samples

*In vitro* assaying of IL-8 cytokine release was performed by seeding HaCaT cells at a density of $3\times 10^{4}$ cells/cm^2^ and treated as previously described. Conditioned medium was collected at the respective timepoints (see 2.6) and tested by means of a Human IL-8 ELISA Kit (abcam) following the instructions of the manufacturer. The medium samples were centrifuged at 2000 × g for 10 min and later diluted 1:10 with Sample Diluent NS included in the kit. The optical density (OD) was measured at a wavelength of 450 nm using a Tecan Infinite M200 plate reader.

### 2.10 Cell staining of CLDN1 and confocal microscopy

Immunofluorescence analysis of CLDN1 protein was performed on HaCaT cells seeded on 12 mm diameter cover slips of 1.5H (0.17 ± 0.005 mm) thickness (Roth) at a density of $3\times 10^{4}$ cells/cm^2^ and treated for 6 and 48 h as previously mentioned. Cells were washed with PBS and fixed with a 3.6% (v/v) formaldehyde in PBS solution for a total time of 25 min. The cells were then washed with PBS again, permeabilized for 20 min with 0.5% Tween20 solution and washed three times with PBS for 5 min each. Subsequent blocking was done with blocking solution (5% (v/v) donkey serum and 0.5% (v/v) triton X-100 (Roth) in SuperBlock buffer (Thermo Fisher Scientific)) for 30 min followed by three washing steps with PBS, again for 5 min each. The labelling of the cells was then done with recombinant anti-CLDN1 antibody (abcam, ab211737) 1:100 (v/v) in SuperBlock buffer with 5% (v/v) donkey serum and 0.2% (v/v) triton X-100. The cells were incubated with this solution for 1.5 h at room temperature (RT) and the primary antibody was thereafter washed away from the cells using PBS in three washing steps (for 5 min each). The next step was the labelling with the secondary antibody by incubation with Alexa Fluor-488-labelled goat-anti-rabbit IgG (Thermo Fisher Scientific, A11008) 1:200 (v/v) for further 1.5 h. Finally, the cells were washed with PBS for 5 min three times and embedded in anti-fade fluorescence mounting medium (abcam) and analysed using confocal laser-scanning microscopy (LSM 800 KMAT, Zeiss). Confocal z stacks were collected with a 63× oil immersion objective. All z-stacks were maintained at a x y pixel distribution of 512 × 512. The acquisition and processing of all the experiment images was performed using representative areas and the same settings. Control staining of cells without the primary antibody were taken for quality assessment of the staining (Supplementary Figure S1). Protein abundance was calculated using ImageJ by selecting the region of interest (ROI) of every cell and calculating the corrected total cell fluorescence (CTCF) against the image background (Gavet and Pines, 2010).

### 2.11 Data analysis

For all statistical analysis Microsoft Excel and GraphPad Prism Version 10.1.1 was used. Data is presented as mean ± standard error of mean (SEM). To verify the normal (Gaussian) distribution of the data different normality tests were performed (D’Agostino-Pearson, Anderson-Darling, Shapiro-Wilk and Kolmogorov-Smirnov normality tests). Additional testing of equal variances was done using either an F-test for comparisons made between two groups or a Brown-Forsythe analysis of variance (ANOVA) test for comparisons between larger data sets. Comparisons made between two groups were analysed using Welch’s t-test for groups with normally distributed data but unequal variances or Mann-Whitney t-test for data sets that did not fulfil either the normality test nor the equal variances requirement. Statistical analysis of larger sets of comparisons were done by means of Brown-Forsythe and Welch ANOVA in combination with Dunnett’s T3 multiple comparisons *post hoc* test for normally distributed data sets with unequal variances or Kruskal-Wallis rank sum test for in combination with Tukey’s multiple comparison *post hoc* test for comparisons between groups that did not fulfil the normality requirement. Outliers were determined and excluded using a Nalimov test. *P*-values of less than 0.05 were considered significant.

## 3 Results

### 3.1 Capsaicin reduced permeability of the skin in an *ex vivo* model

**FIGURE 1:**
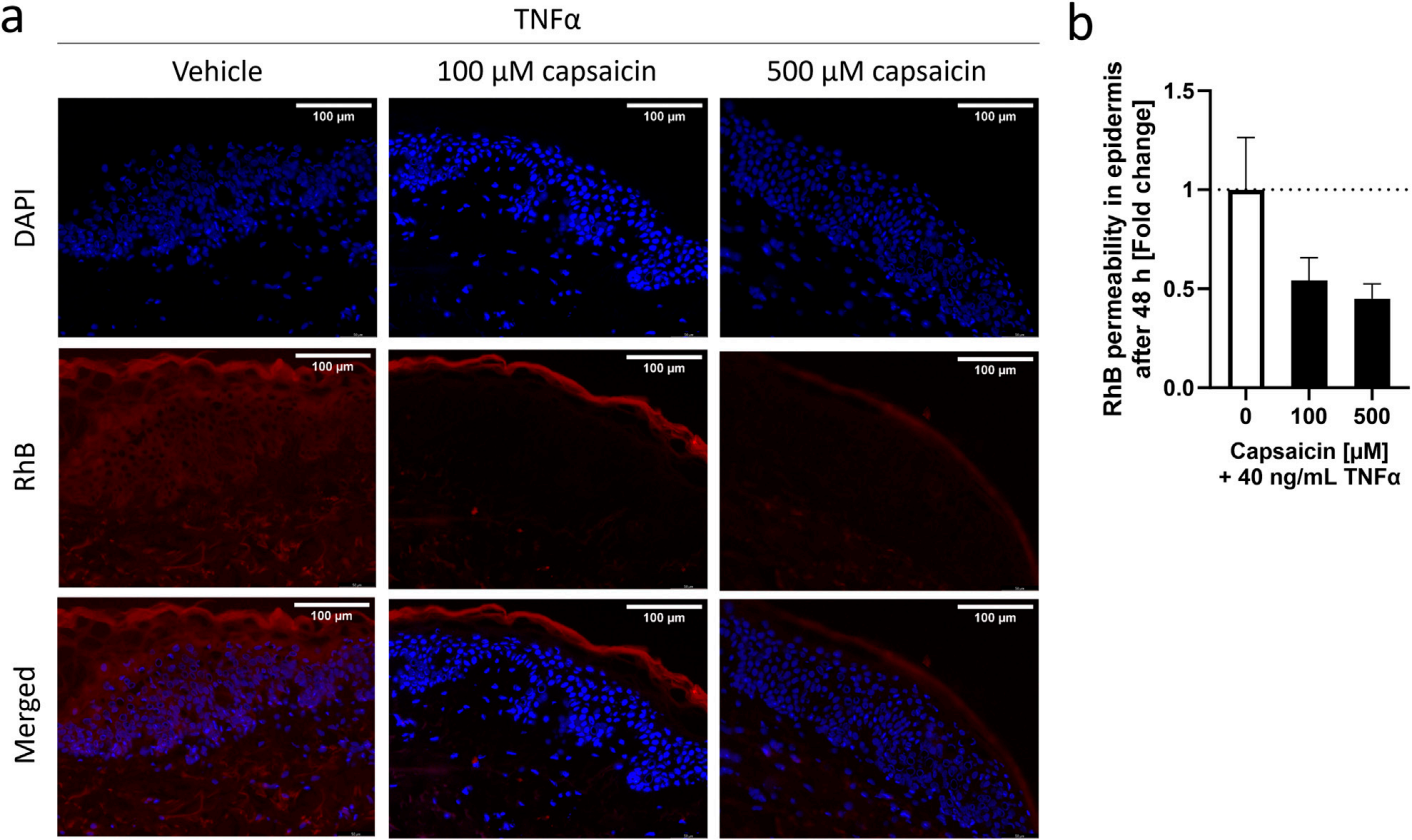
Characterization of capsaicin pre-treatment of skin biopsies and its effect on TNFα-induced inflammation. Epidermal explants of a healthy adult woman, pre-treated with 100 μM, 500 µM or without capsaicin for 24 h, were incubated with 40 ng/mL TNFα for 48 h before measuring the permeability of a RhB dye through the *stratum corneum* (SC) using fluorescence microscopy. **(A)** Images show the permeability of the RhB dye on skin biopsies exposed to vehicle or different concentrations of capsaicin before TNFα-treatment into the inner layers of the epidermis. **(B)** Average fluorescence readings of RhB dye inside the epidermis of capsaicin pre-treated skin biopsies (black) presented as fold change of the non-pretreated control biopsies (white). (Statistics: mean fold change to non-treated control +SEM; technical replicates: 6, biological replicates: 1).

In order to address the effects of capsaicin on the skin and its impact on inflammation, skin biopsies were subjected to a 24 h pre-treatment with two different concentrations of capsaicin, 100 µM and 500 μM, before inducing inflammation with TNFα for a period of 48 h. These capsaicin concentration fall into the “low-concentration” topical treatment range used *in vivo* (Hayman and Kam, 2008; Boyd et al., 2014). TNFα was used at 40 ng/mL as this concentration has been successfully used in HaCaT cells before to induce an inflammatory response (Luostarinen et al., 2021). The selected biomarker for this study is IL-8, as it is a robust inflammatory marker commonly used in skin disease research (Steinhoff et al., 2010; Qiu et al., 2023; Patel et al., 2018). The resulting permeability of the epidermis in the skin biopsy was determined using a RhB dye after inflammation was confirmed by measuring the IL-8 release into the surrounding medium (Supplementary Figure S2). In samples pre-treated with 100 or 500 µM capsaicin, the RhB dye concentrated at the SC, not being able to infiltrate the living cell layers of the epidermis. In absence of capsaicin, the dye could be seen widely distributed in the inner layers of the epidermis hinting to increased permeability after the TNFα treatment for 48 h (Figure 1A). The intensity of RhB fluorescence within the epidermal layer was calculated as the fold change to the TNFα only control and can be seen in (Figure 1B). The permeability of skin biopsies pre-treated with 100 µM capsaicin had a 0.54 ± 0.12-fold decrease in comparison to the non pre-treated TNFα control whereas the permeability of biopsies treated with 500 µM decreased 0.45 ± 0.07-fold. This suggests that the permeability of RhB into the living cell layer of the TNFα treated skin biopsies used in this experiment is reduced approximately 50% if there is a topical pre-treatment with capsaicin.

### 3.2 TNFα promoted IL-8 release and *CLDN1* gene expression in a concentration dependent manner in a basal keratinocyte model

The reduced permeability seen in *ex vivo* settings led to further investigate the effects of capsaicin pre-treatment in an *in vitro* model for skin inflammation. First, we attempted to recreate two different keratinocyte morphologies by varying the calcium concentration in the medium. HaCaT keratinocytes were cultured in medium containing 0.06 mM Ca^2+^ (i.e., low calcium) or 1.8 mM Ca^2+^ (i.e., high calcium). The gene expression data of *CLDN1*, *CLDN2* and *OCLN* are listed in Table 2, showing that the gene expression of *CLDN1* was significantly higher in relation to that of the other two TJ proteins in low and high calcium culture conditions. It was then proceeded to establish a HaCaT inflammation model, treating the cells with 20 ng/mL TNFα under low and high calcium culture conditions. In low calcium condition, TNFα produced a robust 20.54 ± 6.61-fold increase in *CXCL8* gene expression and a 3.51 ± 0.55-fold increase in *CLDN1* gene expression compared to the control. In high calcium conditions TNFα produced 37.40 ± 7.93 fold change in IL-8 release and a comparably lower *CLDN1* fold change increase of 1.31 ± 0.09 (Supplementary Figure S3). The gene expression profile of *CLDN2* and *OCLN* corresponded to the expression profile of lower layer epidermal keratinocytes (Katja Bäsler et al., 2016; Bäsler and Brandner, 2017). *IL6* showed a lower inducibility by TNFα in direct comparison to *CXCL8* under low- and high calcium condition (Supplementary Figure S4). This demonstrated that low calcium culture conditions are suitable to analyze TNFα-induced regulation of CLDN1 and this condition was thus selected to address TJ protein changes after TNFα induction.

**TABLE 2:** Gene expression of different TJ proteins in HaCaT keratinocytes cultured in medium containing 0.06 mM Ca^2+^ or 1.80 mM Ca^2+^.

Gene	0.06 mM Ca^2+^	1.80 mM Ca^2+^
*CLDN1*	0.283 ± 0.042	0.395 ± 0.036
*CLDN2*	0.001 ± 0.0001	0.003 ± 0.0009
*OCLN*	0.020 ± 0.002	0.087 ± 0.006

Data normalized to the geometric mean of the reference genes, *GAPDH*, and *HPRT1*. Data presented as mean ± SEM; technical replicates: 3, biological replicates: 3.

**FIGURE 2:**
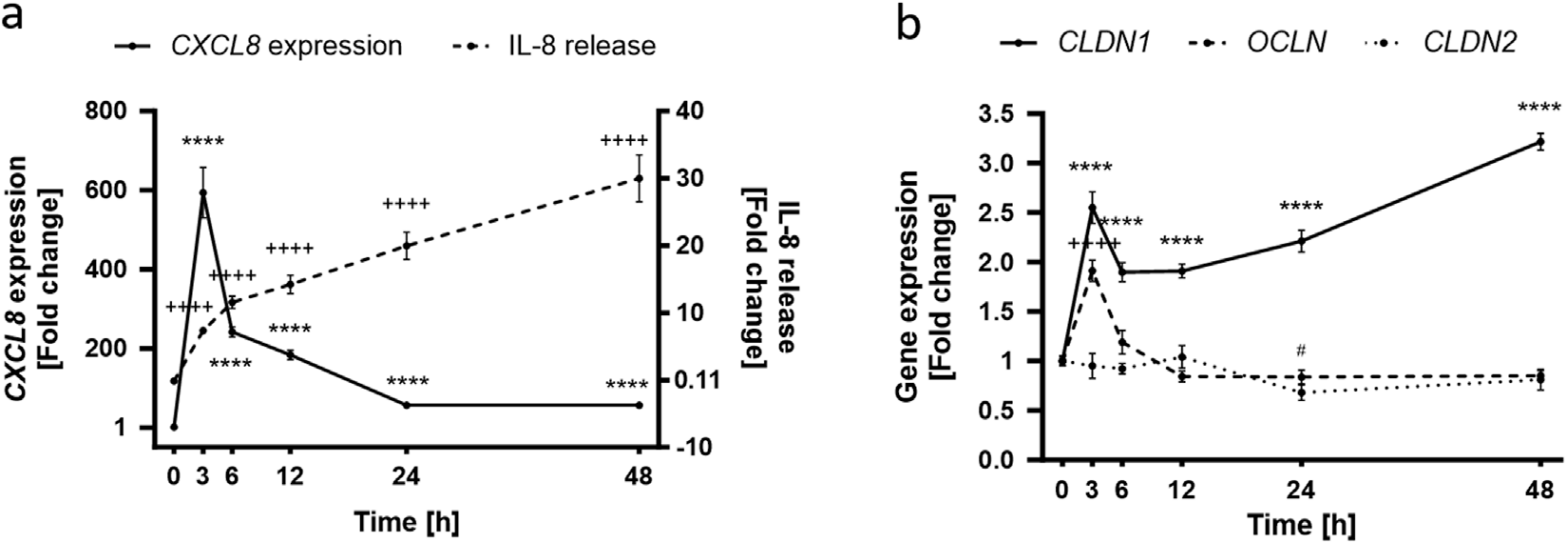
Time dependence of the TNFα-induced inflammatory response in HaCaT keratinocytes. **(A)** TNFα-induced *CXCL8* gene expression peaks at early time points (solid line), subsiding with time which contrasts with the steadily increasing IL-8 release (dashed line) that reaches a maximum at the 48 h time point. Control values for *CXCL8* and IL-8 release are 1.00 ± 0.06 and 0.11 ± 0.05 pg/mL respectively **(B)** *OCLN* expression (dashed line) is significantly increased at early stages of the TNFα response but returns to baseline levels with time. *CLDN1* gene expression (solid line) remains steadily upregulated over the whole course of the measurement seeing an increase at 48 h. *CLDN2* expression (dotted line) is rather unaffected for the first 24 h to see a significant decrease thereafter. Control values for *OCLN*, *CLDN1* and *CLDN2* are 1.00 ± 0.05, 1.00 ± 0.05 and 1.00 ± 0.03, respectively. *CXCL8*, *OCLN*, *CLDN1* and *CLDN2* gene expression were measured using RT-qPCR and IL-8 release was measured using ELISA. (Statistics: mean ± SEM; technical replicates: 3-6, biological replicates: 3-5; Brown-Forsythe and Welch ANOVA with Dunnett’s T3 multiple comparisons *post hoc* test, **(A)** ****p < 0.0001 for significance in *CXCL8* expression (solid line) and ^++++^ *p* < 0.0001 for significance in the IL-8 release (dashed line); **(B)** ****p < 0.0001 for significance in *CLDN1* expression (solid line), ^++++^ *p* < 0.0001 for significance in *OCLN* expression (dashed line), ^#^ *p* < 0.5 for significance in *CLDN2* expression (dotted line).

Because the inflammatory response develops in different stages (Steinhoff et al., 2010), the next step was the characterization of the inflammation model by following the effects of TNFα in HaCaT keratinocytes through a total period of 48 h (Supplementary Figure S5). *CXCL8* expression was significantly increased compared to the non-treated control (1.00 ± 0.06) over the whole 48 h period, reaching a maximum fold change increase of 594.10 ± 63.54 after 3 h (Figure 2A). Thereafter *CXCL8* gene expression subsided until it reached a minimum of 56.13 ± 3.79 at the 48 h time point. IL-8 release, on the other hand, rose steadily over the whole 48 h period, reaching its maximum value at 48 h with a concentration of 30.01 ± 3.49 pg/mL whilst the release in non-treated cells was only 0.11 ± 0.05 pg/mL (Figure 2A). The TJ protein expression over time can be seen in Figure 2B. *CLDN1* was significantly upregulated during the 48 h period by a fold increase between 2.60 ± 0.16 (3 h) and 3.22 ± 0.09 (48 h) compared to non-treated control values (1.00 ± 0.03) (Figure 2B). This constant upregulation was not seen for the other two TJ proteins analyzed. *OCLN* gene expression increased significantly at 3 h to a 1.91 ± 0.11-fold increase but returned to baseline control values (1.00 ± 0.05) thereafter and *CLDN2* expression was not increased but was significantly reduced at 24 h by 0.83 ± 0.07-fold as compared to the non-treated control (1.00 ± 0.05) (Figure 2B). A steady upregulation of *CXCL8* and consequent IL-8 release was induced by TNFα treatment and this was accompanied by the constant upregulation of *CLDN1* during a measurement period of 48 h. TJ proteins *OCLN* and *CLDN2* were, in contrast, transiently up or downregulated after TNFα treatment.

**FIGURE 3:**
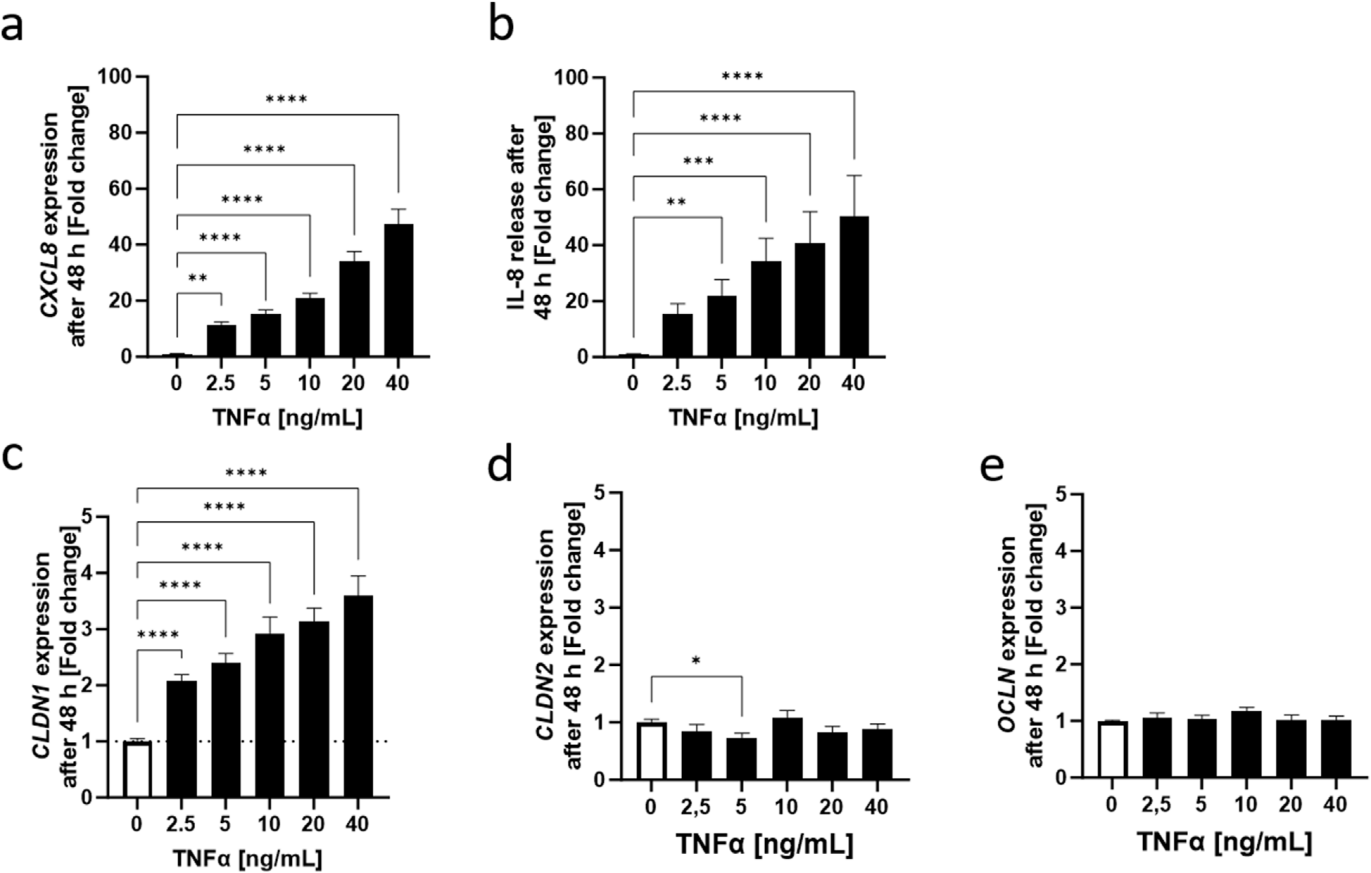
Increasing concentrations of TNFα promote **(A)** *CXCL8* gene expression and **(B)** IL-8 release after an incubation period of 48 h. This is accompanied by an equally concentration dependent increase in **(C)** *CLDN1* gene expression. Expression of **(D)** *CLDN2* does not show a concentration dependent increase but is rather stable during the 48 h period with the exception of significant downregulation using 5 ng/mL TNFα. **(E)** Expression of *OCLN* was similar to the control over all concentrations. *CXCL8, CLDN1, CLDN2* and *OCLN* gene expression were measured using RT-qPCR and IL-8 release was measured using ELISA. Data is presented as fold change to non-treated control. White bars represent the non-treated control and black bars represent TNFα treatment at different concentrations. (Statistics: mean +SEM; technical replicates: 3-6, biological replicates: 3-5; **(A–B)** Kruskal Wallis test with Dunn’s multiple comparisons *post hoc* test **p < 0.01, ***p < 0.001, ****p < 0.0001; **(C–E)** Brown-Forsythe and Welch ANOVA with Dunnett’s T3 multiple comparisons *post hoc* test, *p < 0.05, ****p < 0.0001).

Following the establishment of a time profile for the effect of TNFα in HaCaT keratinocytes, the dose dependency of the keratinocyte response to TNFα was determined using TNFα at concentrations ranging from 2.5 to 40 ng/mL (Supplementary Figure S5). The maximum concentration used *in vitro* was the same concentration used *ex vivo* to establish inflammation. Reduction of cell viability by TNFα was excluded by means of an MTT assay (not shown). Already at a TNFα concentration of 2.5 ng/mL, a 11.25 ± 1.05-fold increase in *CXCL8* expression could be seen compared to the values of non-treated control cells. Increasing TNFα concentrations led to increasing *CXCL8* expression levels and the maximum measured values were reached with 40 ng/mL TNFα with 47.39 ± 5.36-fold change to control (Figure 3A). IL-8 release was also significantly increased over the whole concentration range with 15.55 ± 3.60-fold increase at the lowest TNFα concentration and a maximum 50.48 ± 14.45-fold increase in the release at the maximum TNFα concentration (Figure 3B). Interestingly, the dose-dependent gene expression profile of the TJ proteins *CLDN1*, *CLDN2* and *OCLN* differed from each other significantly. There is a steep upregulation of *CLDN1* over the whole measured range of concentrations. At 2.5 ng/mL TNFα a 2.08 ± 0.11-fold change increase was observed, that increments with higher concentrations until 40 ng/mL TNFα with a 3.60 ± 0.36-fold change to the non-treated control (Figure 3C). *CLDN2* and *OCLN*, on the other hand, did not share this concentration-dependent increase. *CLDN2* gene expression was downregulated only when using a TNFα concentration of 5 ng/mL (0.74 ± 0.08-fold change) compared to control values (Figure 3D). *OCLN* was not significantly different from control values at any of the concentrations (Figure 3E).

**FIGURE 4:**
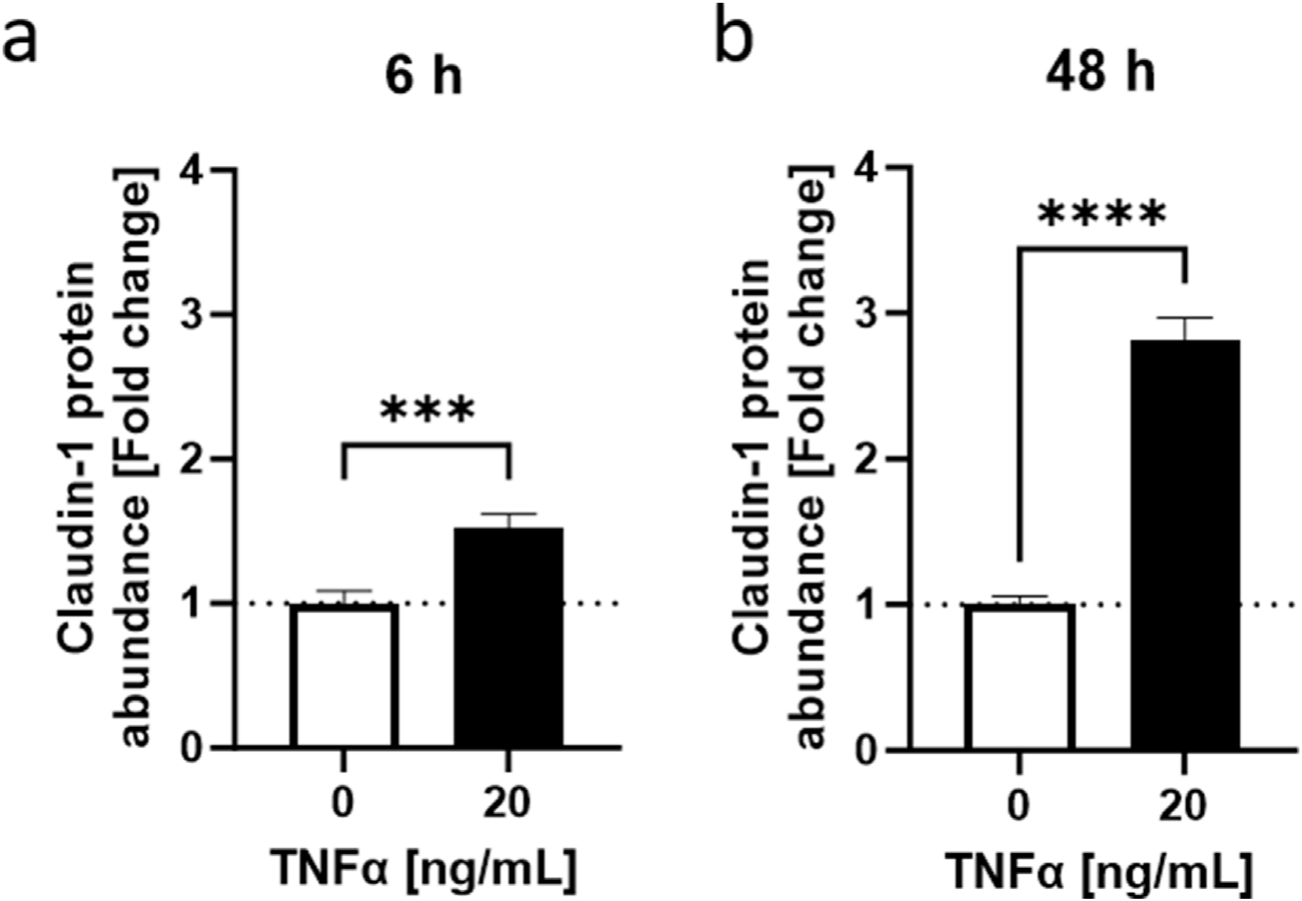
TNFα-induced increase in CLDN1 protein abundance in HaCaT keratinocytes. TNFα increased claudin-1 protein abundance at **(A)** 6 h and **(B)** 48 h. Claudin-1 protein abundance was measured with fluorescence microscopy. Data is presented as fold change to non-treated control. White bars represent the non-treated control and black bars represent TNFα treatment at different concentrations. (Statistics: mean +SEM; technical replicates: 3-6, biological replicates: 3-5; Mann-Whitney t-test, ***p < 0.001, ****p < 0.0001).

To summarize, the HaCaT inflammation model under low calcium conditions demonstrated that TNFα induced a response over a wide range of concentrations and time points which significantly impacted *CLDN1* gene expression and the cytokine IL-8 on gene and protein level, respectively. To further characterize the effect of TNFα in HaCaT keratinocytes, we decided to focus on the expression and protein abundance of CLDN1 using 20 ng/mL TNFα as the stimulus concentration. It was also decided to address the 6 and 48 h time points in order to separate early and later stages of the inflammatory response. As TNFα promoted the gene expression of *CLDN1* in keratinocytes for 48 h and this could eventually lead to an accumulation of CLDN1 protein in the cells we tested cells treated with TNFα for short (6 h) and long (48 h) periods of time and the protein amount of CLDN1 was measured using fluorescence microscopy. The calculated abundance of CLDN1 protein was in both time points significantly higher to that of control samples without TNFα. After 6 h, there was a 1.52 ± 0.10-fold increase (Figure 4A) and after 48 h a 2.81 ± 0.16 fold increase (Figure 4B) compared to the control values. CLDN1 protein accumulated, thus, in HaCaT keratinocytes that simulate the morphology of keratinocytes in lower epidermal layers after TNFα exposure.

### 3.3 Capsaicin reduced TNFα-dependent IL-8 release and promoted *CLDN1* gene expression after 6 h

**FIGURE 5:**
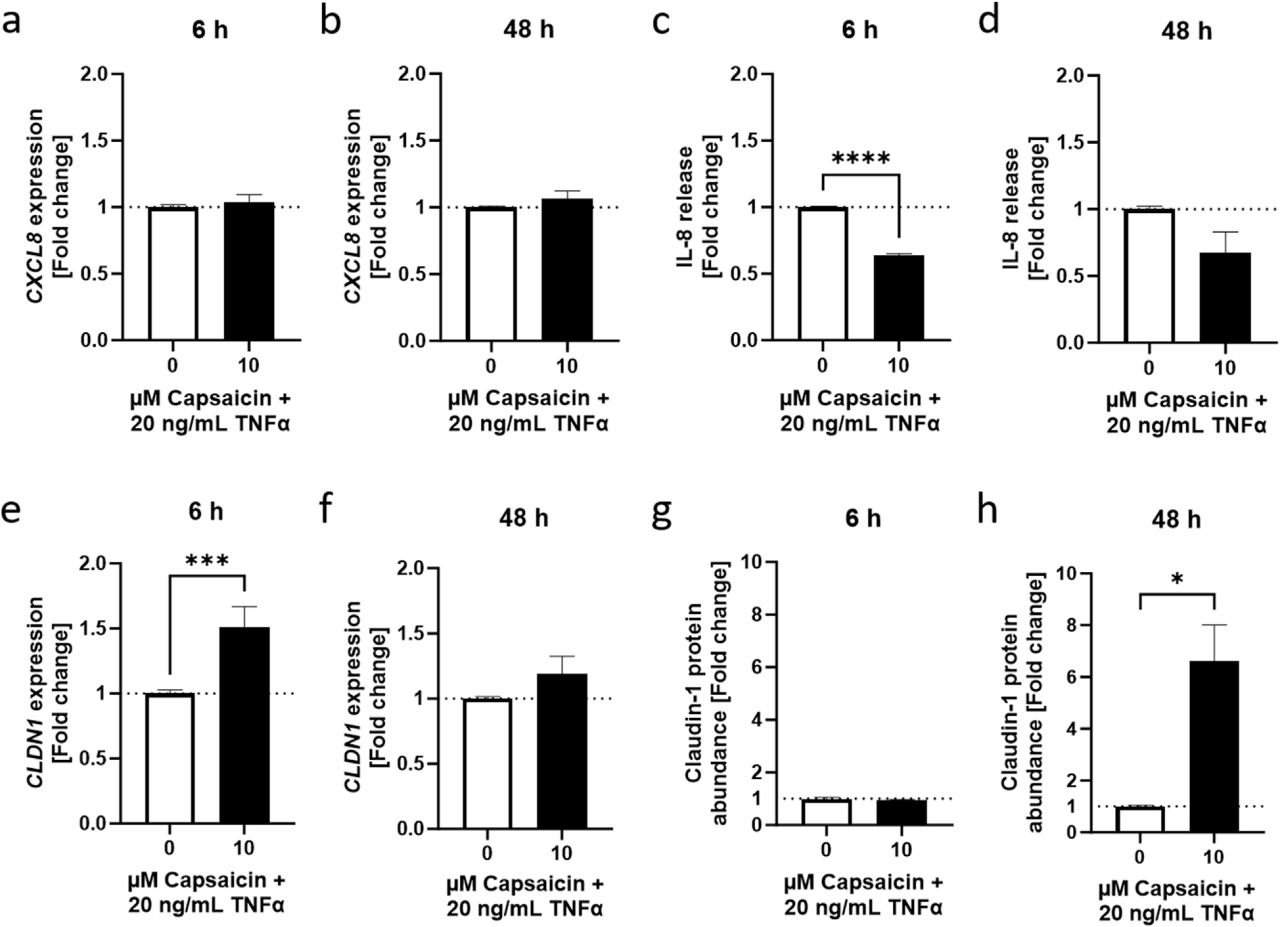
Capsaicin regulates the TNFα-induced inflammatory response in HaCaT keratinocytes. **(A, B)** Cells pretreated with 10 µM capsaicin for 24 h did not show any modulation of the TNFα response with regards to *CXCL8* gene expression but **(C, D)** IL-8 release was significantly attenuated after 6 h and remained lower than in cells that were not pre-treated over 48 h **(E, F)** *CLDN1* gene expression was significantly enhanced in pre-treated cells 6 h after start of the TNFα treatment but the effect subsided with time. **(G, H)** An increase of CLDN1 protein abundance was evident after 48 h in comparison to the non pre-treated control. *CXCL8* and *CLDN1* gene expression obtained with real-time PCR and IL-8 release using ELISA. CLDN1 protein was imaged with fluorescence microscopy and fluorescence intensity calculated as fold change of the control. Data presented as the fold change to the non pre-treated TNFα control. (Statistics: mean +SEM; technical replicates: 3, biological replicates: 3; **(A–D, F)** Welch’s t-test, ****p < 0.0001; **(E, G, H)** Mann-Whitney t-test, *p < 0.05, ***p < 0.001).

Next, the effect of capsaicin pretreatment on CLDN1 in the inflammation model for HaCaT of the lower epidermal layers was determined. As expected, cellular toxicity of capsaicin in combination with capsazepine allowed only lower concentrations of capsaicin to be used than in the *ex vivo* model, finding the optimum to be 10 µM using MTT assays (not shown). This concentration also showed an anti-inflammatory role of capsaicin in macrophages *in vitro* (Walker et al., 2017). HaCaT keratinocytes were incubated with 10 µM capsaicin for 24 h before treatment with 20 ng/mL TNFα for 6 or 48 h. The effect size of the capsaicin pre-treatment without subsequent TNFα stimulation was negligible when compared to TNFα treated equivalents (Supplementary Figure S6). *CXCL8* expression was not significantly altered by the capsaicin pre-treatment (Figures 5A, B). IL-8 release was significantly reduced 0.64 ± 0.017-fold at the 6 h time point compared to samples that were only subject to the TNFα treatment (Figure 5C). This effect was not as pronounced in samples incubated for 48 h (Figure 5D). The short-term impact of a 24 h capsaicin treatment attenuated, therefore, the release of IL-8 into the medium 6 h after induction with TNFα.

The 24 h pre-treatment of the HaCaT keratinocytes with capsaicin also affected *CLDN1* gene expression. There was a 1.51 ± 0.16-fold change increase during the first 6 h in samples that were pre-treated with capsaicin compared to samples that did not undergo pre-treatment before induction with TNFα (Figure 5E). Later, at 48 h, the upregulation of *CLDN1* relative to the values of the TNFα treated control was no longer present (Figure 5F). CLDN1 protein abundance was not significantly increased in capsaicin pre-treated samples 6 h after TNFα induction (Figure 5G) but further analysis of the 48 h time point after incubation with TNFα showed a 6.61 ± 1.40-fold increase in CLDN1 protein abundance of cells pre-treated with capsaicin compared to cells treated only with TNFα (Figure 5H). Thus, the increased *CLDN1* gene expression in earlier time points of the inflammatory reaction could contribute to increased production of CLDN1 protein, which was seen at later stages of the response.

**FIGURE 6:**
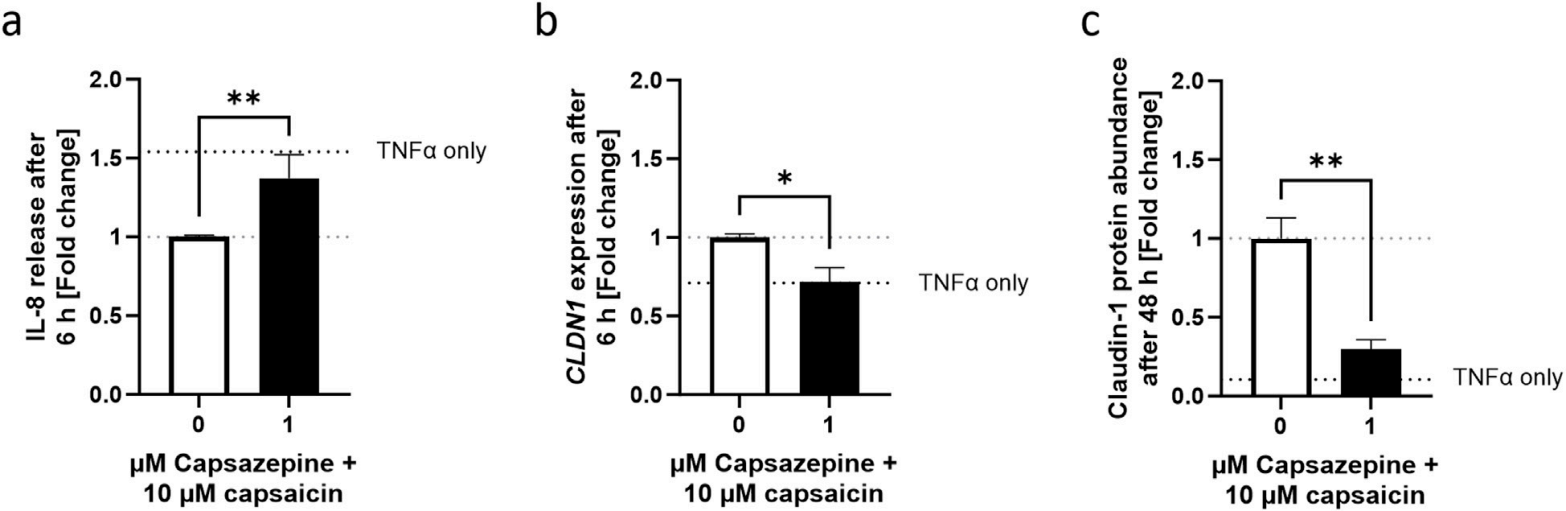
Decreased IL-8 release and promotion of CLDN1 after capsaicin treatment is mediated by the TRPV1 channel. **(A)** Cells pre-treated with capsaicin for 24 h reduced the IL-8 release during the first 6 h of TNFα-induced inflammation. Co-incubation with 1 µM capsazepine during the pre-treatment prevented the attenuation of the IL-8 release. **(B, C)** Similarly, the promotion of *CLDN1* and increased abundance of CLDN1 seen after pre-treatment with 10 µM capsaicin at 6 and 48 h, respectively, was no longer present when 1 µM capsazepine was added in the pre-treatment. IL-8 release assessment was done using ELISA, *CLDN1* gene expression measured using real-time PCR and CLDN1 protein was measured with fluorescence microscopy. Data is presented as fold change to capsaicin treated control. The dotted black line shows the effect elicited by TNFα without pre-treatment. (Statistics: mean +SEM; technical replicates: 3, biological replicates: 3; **(A, C)** Mann-Whitney t-test, **p < 0.01; **(B)** Welch’s t-test, *p < 0.05).

To verify whether the keratinocyte TRPV1 receptor is involved in the mediation of the effects of capsaicin, a well-known TRPV1 inhibitor, capsazepine (Walker et al., 2003), was used in combination with capsaicin. In cells pre-treated with the capsaicin-capsazepine combination, IL-8 release reached values as high as those of cells that only received a 6 h long TNFα treatment, thus reversing the anti-inflammatory effect seen in cells pre-treated only with capsaicin (Figure 6A). Similarly, *CLDN1* gene expression was brought back down to TNFα control levels (Figure 6B) and CLDN1 protein abundance at 48 h remained as low as in samples only treated with TNFα (Figure 6C). This suggests that the TRPV1 channel is involved in the effects evoked by the capsaicin pre-treatment over the course of the inflammatory reaction induced by TNFα.

### 3.4 Capsaicin pre-treatment promoted relocation of CLDN1 protein towards the cell boundaries after 6 h and 48 h

**FIGURE 7:**
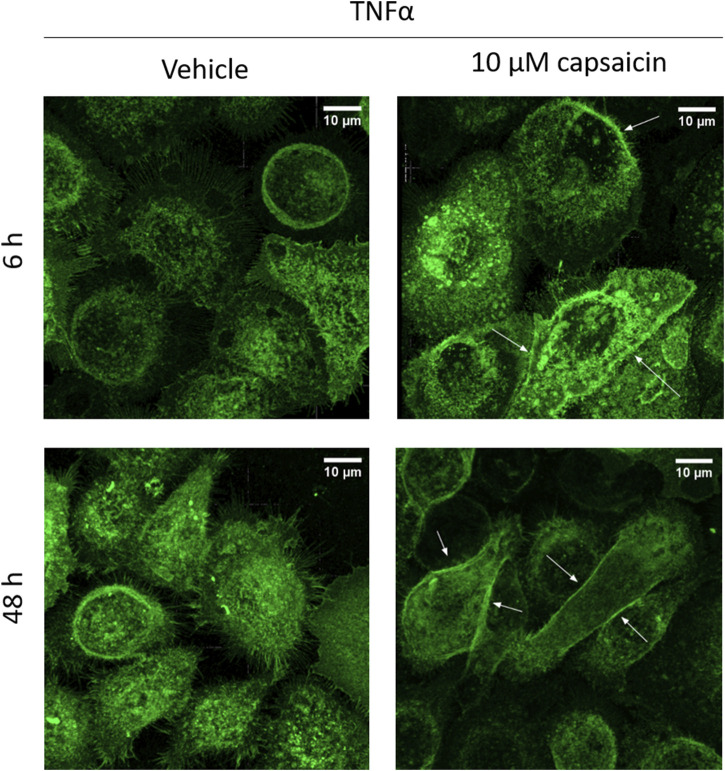
CLDN1 accumulates in cell boundaries after capsaicin pre-treatment. HaCaT cells that were pre-treated for 24 h with 10 µM capsaicin before 20 ng/mL TNFα treatment showed increased positioning of CLDN1 protein close to the boundaries of the cell when compared to cells treated solely with TNFα (arrows). Already at 6 h, there is definition of the cell boundaries between the keratinocytes. This effect is further enhanced at 48 h, with CLDN1 seemingly redistributing from the inner part of the cell to the boundaries.

The increase of CLDN1 protein seen at 48 h suggests that an early stimulation of its gene expression would develop into more presence of protein in later time points. This is consistent with the decreased permeability seen *ex vivo*. However, not only the amount of CLDN1 in the cells was increased when using capsaicin as a pre-treatment, but also the positioning CLDN1 protein towards the cell borders was promoted (Figure 7). In capsaicin pre-treated cells, there was migration of the protein towards the cell boundaries already 6 h after induction by TNFα. This migration was more evident at the 48 h timepoint, where the cells accumulated and exhibited more defined junctions in the paracellular space. This effect was not observed in cells that did not undergo capsaicin treatment prior to TNFα addition. Non pre-treated cells still not define any boundaries between adjacent cells but rather have a wider distribution of CLDN1 in the inner space. This further corroborates the hypothesis that capsaicin impacts the production and functionality of CLDN1 and its relation to permeability.

## 4 Discussion

In this study we address the effects of capsaicin on skin cells during TNFα-induced inflammation. Capsaicin is used against chronic pain and pruritus, yet also acts as a skin irritant (Boyd et al., 2014). Nonetheless, recent studies have pointed at an anti-inflammatory and barrier recovery effect of capsaicin on epithelial tissues (Zhao et al., 2021). Activation of the TRPV1 channel by capsaicin, therefore, has the potential to reduce inflammatory markers and promote keratinocyte barrier development. Because current *in vitro* models do not investigate the effect of capsaicin stimulation of keratinocyte TRPV1 channels on barrier function in inflammation, an inflammatory model of cultured keratinocytes was created. This model focuses on lower epidermal layers (i.e., SB or SS) where TJs are not yet formed and therefore the initial stages of barrier formation during inflammation can be researched. In comparison with previous studies that focused on capsaicin in murine models of barrier recovery after tape stripping (Yun et al., 2011; Denda et al., 2007) or models focused on neuronal TRPV1 activation (Del Bianco et al., 1996; Newbold and Brain, 1995; Krogstad et al., 1999), this model offers a unique perspective on the role of keratinocyte TRPV1 channels in the skin. Combined with *ex vivo* experiments on epidermal permeability, a comprehensive picture of the barrier function of keratinocytes in the epidermis can be drawn.

First, the effect of capsaicin treatment in barrier permeability during inflammation was characterized *ex vivo*. We evaluated whether a 24 h pre-treatment with commercially used concentrations of capsaicin would have a negative effect on the permeability of the epidermis in skin biopsy samples that were subjected to the inflammatory cytokine TNFα for 48 h. In the skin biopsy which received 100 µM or 500 µM capsaicin-treatments a reduced permeability in comparison to the ones that were pre-treated only with the vehicle DMSO was demonstrated. Thus, the results show a reduced permeability of the epidermis in the *ex vivo* biopsies that receive a 24 h application of capsaicin before being exposed to the effects of TNFα.

To investigate the effect of capsaicin pre-treatment on the permeability observed in the *ex vivo* experiment, it was decided to develop an inflammation model based on HaCaT keratinocytes. Through variations of the calcium concentration in the medium, two types of keratinocyte morphologies in the epidermis were modelled. Under low calcium conditions (0.06 mM Ca^2+^) keratinocytes are rounded, cubical cells that are separated by a gap. This morphology mimics that of SB or SS keratinocytes that not yet transitioned to the flattened, tightly packed formation characteristic of cells in the SG, the outermost layer of the living-cell epidermis (Wilson, 2014). The typical characteristics of SG keratinocytes could be modelled by high calcium (1.8 mM Ca^2+^) culture conditions. Because TJs are determinant for the permeability in the epidermis, there was an interest to see how the gene expression profile of TJ proteins was affected by TNFα in low vs. high calcium conditions. The gene expression of three essential TJ proteins, CLDN1, CLDN2 and OCLN was congruent with the effect of TNFα on TJs in other tissues (Iida et al., 2022; Amoozadeh et al., 2015; Zhang et al., 2019). For this reason, low calcium culture conditions were selected and a larger effect size of the TNFα treatment with regards to TJ protein expression was confirmed.

The inflammation model was further characterized by incubating low calcium HaCaT keratinocytes with different concentrations of TNFα for 48 h. The effect observed in other studies involving glomerular and lung epithelia (Iida et al., 2022; Shiozaki et al., 2012) of a TNFα-dependent increase of *CLDN1* gene expression corresponded well with the upregulation of *CLDN1* expression observed after 48 h treatment of HaCaT keratinocytes with TNFα. *CLDN1* upregulation was consistent over a wide range of TNFα concentrations and sustained throughout the 48 h period. This gene expression profile differed significantly to that of *OCLN* and *CLDN2*. OCLN is a TJ stabilizing protein that is limited to the outer layers of the epidermis in healthy skin but shows a wider layer distribution in the epidermis during inflammation (Bäsler and Brandner, 2017). Accordingly, *OCLN* expression would be expected to increase after TNFα treatment of keratinocytes modelling lower layers of the epidermis. Although, there was no dose dependent effect of TNFα on *OCLN* gene expression after 48 h, the transient upregulation of *OCLN* 3 h after TNFα induction points to an early promotion of *OCLN* expression. On the other hand, the gene expression of *CLDN2* was downregulated starting 24 h after TNFα induction and continuing until the 48 h time point which suggests a delayed TNFα-dependent response in the case of this TJ protein, consistent with the time profile of *CLDN2* expression after TNFα action seen in other tissues (Amoozadeh et al., 2015). Nevertheless, the downregulation of *CLDN2* after 48 h was not dose-dependent. Consequently, the gene expression of *CLDN1*, *CLDN2* and *OCLN* is impacted at different stages of the TNFα-induced response in keratinocytes that mimic lower epidermal layer populations. Of these three TJ proteins, only *CLDN1* expression is consistently upregulated during the 48 h measurement period and is also dose dependent on TNFα. The abundance of the CLDN1 protein in the cell was also higher 6 and 48 h after TNFα treatment. It was established that there is a sustained TNFα-dependent promotion of CLDN1 production over 48 h in keratinocytes modelled after lower layers of the skin, which can be effectively monitored in comparison to CLDN2 or OCLN. In the following experiments, a clear distinction between early and late stages of TNFα-induced inflammation was set using the 6 and 48 h timepoints and the changes in CLDN1 evaluated after use of 20 ng/mL TNFα as a treatment concentration.

As the inflammatory model was characterized and the relationship between TNFα and CLDN1 established, capsaicin was then tested at a concentration that had been previously used in macrophages and proven to have anti-inflammatory properties (Walker et al., 2017). The use of capsaicin for 24 h before induction of inflammation with TNFα proved to counteract the IL-8 release provoked by TNFα during the first 6 h, which is considered a rather early stage of the inflammatory reaction (Steinhoff et al., 2010; Hackett et al., 2008). Capsaicin treatment is thought to inhibit phosphorylation of IκBα in early stages of the NF-kB pathway as well as phosphorylation of ERK in the MAPK signaling cascade, thereby preventing the respective transcription factors to promote pro-inflammatory cytokine expression (Shin et al., 2013; Li et al., 2021). This could lead to lower levels of IL-8 release by keratinocytes. This, however, would need to be confirmed in future studies.

During this early stage, the gene expression of *CLDN1* was, on the other hand, upregulated compared to the non pre-treated control. Capsaicin, therefore, not only has an anti-inflammatory property but promotes gene expression of *CLDN1* at acute stages of inflammation. The increased abundance of CLDN1 protein in the cells, however, was observed only after 48 h and it might be a consequence of early stage upregulation of the gene due to the capsaicin treatment. Nonetheless, a redistribution of CLDN1 protein towards the keratinocyte cell boundaries was seen at 6 and 48 h. This could be taken as a step towards a progression of the cells into a differentiation stage where TJs are formed.

Because capsaicin is a well-known TRPV1 agonist (Rosenbaum Emir, 2017) we investigated the involvement of keratinocyte TRPV1 channels in the effects seen by using a combination of capsaicin and capsazepine, a TRPV1 antagonist, as a pre-treatment. The effects evoked by the capsaicin pre-treatment were no longer observed and the levels of IL-8 release as well as *CLDN1* gene expression returned to values similar to those of the cells that did not receive a capsaicin pre-treatment before TNFα induction. The levels of CLDN1 protein in the cells after 48 h TNFα treatment were back to baseline levels as well, thereby confirming the involvement of the keratinocyte TRPV1 channel in the effects seen. Previous studies have shown that TRPV channels are connected to TJ functionality (Reiter et al., 2006). Here we show that activation of keratinocyte TRPV1 channel attenuates the release of inflammatory cytokine IL-8 and enhances *CLDN1* expression induced by TNFα. The promotion of CLDN1 after activation of the TRPV1 channel adds to the hypothesis that TRP channels modulate TJ function in keratinocytes. Additionally, the combined anti-inflammatory and TJ protein promotion is consistent with the protective effects seen in other tissues after the use of capsaicin (Zhao et al., 2021; Qiao et al., 2021), especially regarding epithelial tissues. Further analysis of the effects of capsaicin on a wider range of pro-inflammatory cytokines would complete the picture on capsaicin’s anti-inflammatory potential, however, this highlights the importance of capsaicin in the treatment of inflammatory diseases and the need to develop non-pungent analogs that could take advantage of the positive effects of capsaicin without inducing the adverse effects.

The results shown in this study present an advantageous role of keratinocyte TRPV1 channels in inflammation. However, these results are not without limitations. The capsaicin effect on *ex vivo* skin was analyzed just qualitatively on the tissue of a single donor and needs validation in a larger study setup. *Ex vivo* studies, have the advantage to be the closest preclinical models to *in vivo* situations, but they would require a larger sample size in comparison to *in vitro* models. This is due to the fact that the different donors vary in their geno- and phenotype, consequently the results are susceptible to higher variability than isolated cells. Regarding the *in vitro* keratinocyte model of inflammation, the relationship between TJ proteins and keratinocyte TRPV1 channels is explored in this study which is rarely addressed in skin research. Generally, the focus lies on the barrier function of the SC, albeit a characteristic feature of chronic inflammatory diseases is the impairment of TJ protein function and the decreased expression of TJ proteins (Bergmann et al., 2020; Gruber et al., 2015; Kirschner et al., 2009). Although this study furthers the investigation on the influence of keratinocyte TRPV1 channels on TJs, it must be stated that this model is a simplified version of the keratinocyte participation during inflammation. The time difference in the gene expression of *CLDN1*, *CLDN2* and *OCLN* is an example of the complexity of the system. This complexity is amplified when we take into consideration that this cell type is not isolated and interacts with other cellular components in the epidermis. We cannot exclude participation of other TRP channels or keratinocyte receptors, especially when we take into account the length of the pre-treatment incubation (24 h). Furthermore, the presence and functionality of TRPV1 in HaCaT cells have been documented in previous studies (Huang et al., 2017; Kwon et al., 2018), but the TRPV1 protein content was not evaluated in this study. Nevertheless, this study establishes a precedent for the development of comprehensive *in vitro* models representing the influence of inflammatory mediators on TJ development in different keratinocyte layers.

In conclusion, this study evaluated the effects of capsaicin in the skin barrier in *ex vivo* organotypic cultures and we developed an *in vitro* skin inflammation model that allowed us to characterize the relationship between inflammation marker TNFα and TJ protein CLDN1, as well as the modulation of this relationship by capsaicin. Our findings show that capsaicin reduced the permeability of the epidermis and, not only has an anti-inflammatory effect as demonstrated by the attenuation of IL-8 release, but it also promotes the production of CLDN1 protein in keratinocytes with a marked redistribution of CLDN1 protein towards the cell boundaries. We were also able to confirm the involvement of keratinocyte TRPV1 in these effects. This is particularly important, because in the context of inflammation the focus is directed towards the role of neuronal TRPV1 channels. It is important to mention that, although promising, these results only offer a partial understanding of the complex interplay involved in skin inflammation and barrier permeability. Further advances in this field require the analysis of the effects of capsaicin in co-culture models comprising more cell types involved in inflammation. Our results are regarded as an initial impulse to the development of TRPV1-related treatment for skin inflammatory diseases in the future.

## Data Availability

The original contributions presented in the study are included in the article/Supplementary Material, further inquiries can be directed to the corresponding author.
