# Supplementary material for: Capsaicin attenuates the effect of inflammatory cytokines in a HaCaT cell model for basal keratinocytes
Source: Front Pharmacol. 2024 Oct 14;15:1474898. doi: 10.3389/fphar.2024.1474898 (PMC11513304; doi:10.3389/fphar.2024.1474898)
Supplement: Supplementary file 1 [file DataSheet1.PDF]

Supplementary Material

Capsaicin attenuates the effect of inflammatory cytokines on a HaCaT cell model for basal keratinocytes

Maria Fernanda Cervantes Recalde, Jana Schmidt, Cristina Girardi, Marco Massironi, Markus Rechl, Joachim Hans, Dominik Stuhlmann, Veronika Somoza, Barbara Lieder*

*** Correspondence:** Corresponding Author: Barbara.Lieder@univie.ac.at

1 Supplementary Figures

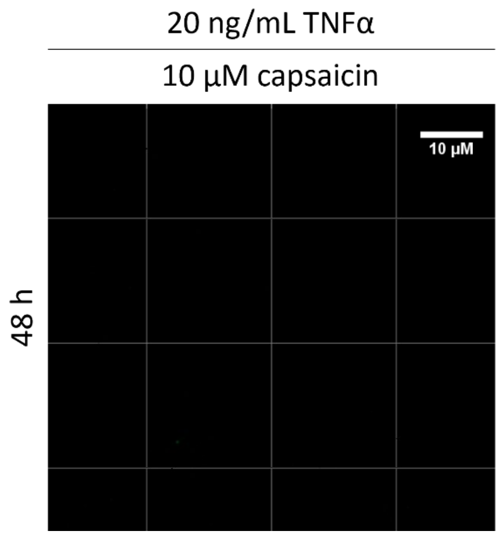

FIGURE S1. Confocal microscopy control staining of cells without the primary antibody. HaCaT cells were pre-treated for 24 h with 10 µM capsaicin followed by 48 h treatment with 20 ng/mL TNFα. Staining was performed as described with the exception of the incubation with recombinant anti-CLDN1 antibody.

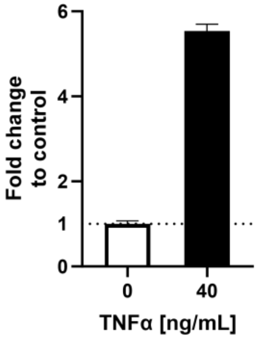

FIGURE S2. Characterization of inflammatory IL-8 release in skin samples after TNFα treatment for 48 h. Epidermal explants of a healthy adult woman, were systemically treated using Williams' Medium E with or without (untreated control) 40 ng/mL TNFα for 48 h. IL-8 release was measured by means of an ELISA assay in medium collected after the 48 h treatment and the result presented as fold change of the non-treated control. (Statistics: mean + SEM; technical replicates: 12, biological replicates: 1)

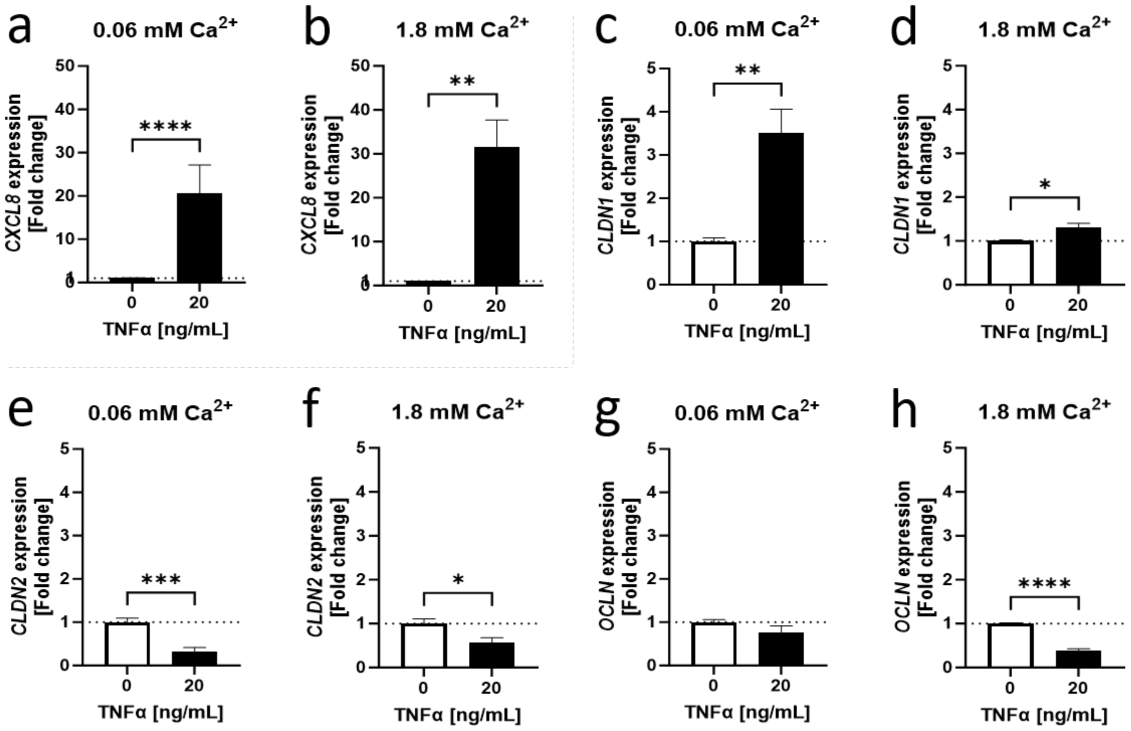

FIGURE S3. Characterization of TNFα induced TJ protein gene expression in HaCaT keratinocytes cultured in low vs. high calcium conditions. HaCaT keratinocytes were cultured in medium containing 0.06 mM Ca^{2+} or 1.80 mM Ca^{2+} for 4 days and then treated with 20 ng/mL TNFα for 48 h. (a, b) *CXCL8*, (c, d) *CLDN1*, (e, f) *CLDN2* and (g, h) *OCLN* gene expression after TNFα treatment (black) presented as fold change of the non-treated control (white). Relative gene expression of the different genes was measured with RT-qPCR and normalized to the geomean of the reference genes (i.e. *GAPDH* and *HPRT1*). (Statistics: mean + SEM; technical replicates: 3, biological replicates: 3, (a, e) Mann Whitney t-test, (b, c, d, g, h) Welch's t-test or (f) unpaired t-test , *p<0.05, **p<0.01, ***p<0.001, ****p<0.0001)

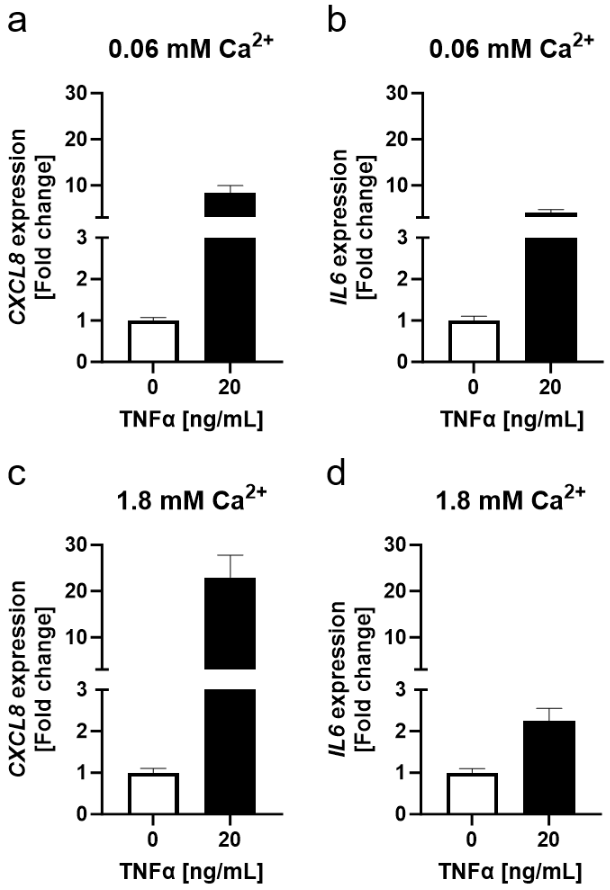

Figure S4. Characterization of *CXCL8* and *IL6* gene expression in HaCaT keratinocytes cultured in low vs. high calcium conditions. HaCaT keratinocytes were cultured in medium containing 0.06 mM Ca^{2+} or 1.80 mM Ca^{2+} for 4 days and then treated with or without 20 ng/mL TNFα for 48 h. *CXCL8* and *IL6* gene expression after TNFα treatment (black) presented as fold change of the non-treated control (white). Relative gene expression of the different genes was measured with RT-qPCR and normalized to the reference gene *GAPDH*. (Statistics: mean + SEM; technical replicates: 3, biological replicates: 1)

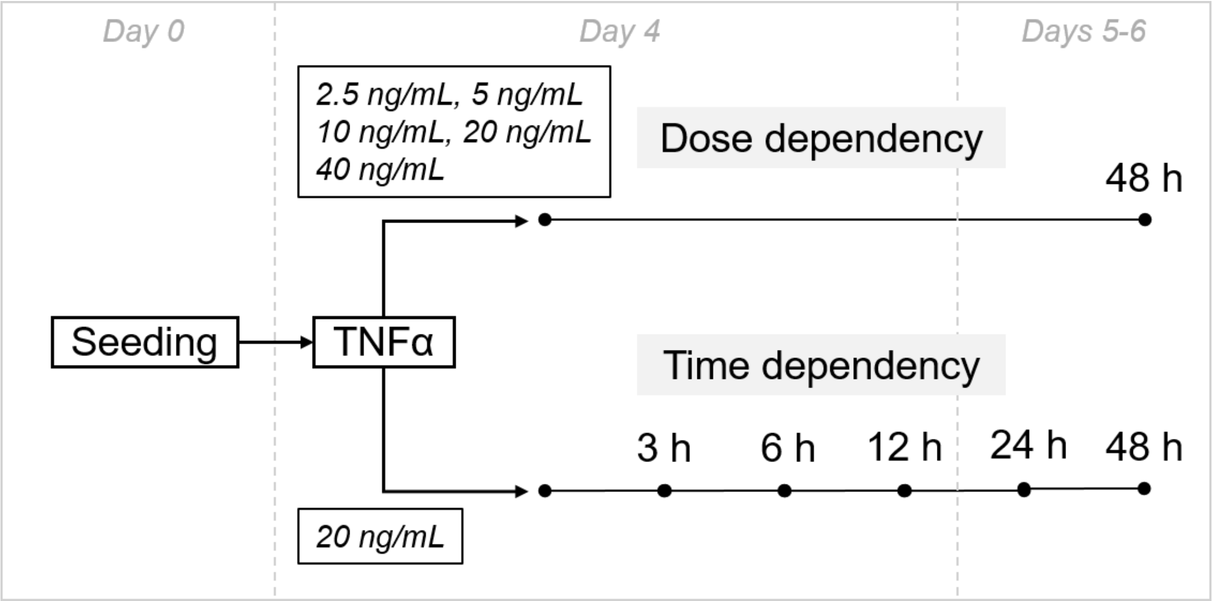

FIGURE S5. TNFα treatment scheme for dose and time dependency of HaCaT keratinocytes. HaCaT keratinocytes seeded under low calcium conditions (0.06 mM Ca^{2+}) were treated with different concentrations of TNFα for a period of 48 h or with 20 ng/mL for 3, 6, 12, 24 and 48 h. Samples were taken at the mentioned time points for RNA isolation, RT-qPCR of the genes *CXCL8*, *CLDN1*, *CLDN2*, *OCLN*, *GAPDH* and *HPRT1* as well as to evaluate IL-8 cytokine release by means of an ELISA assay.

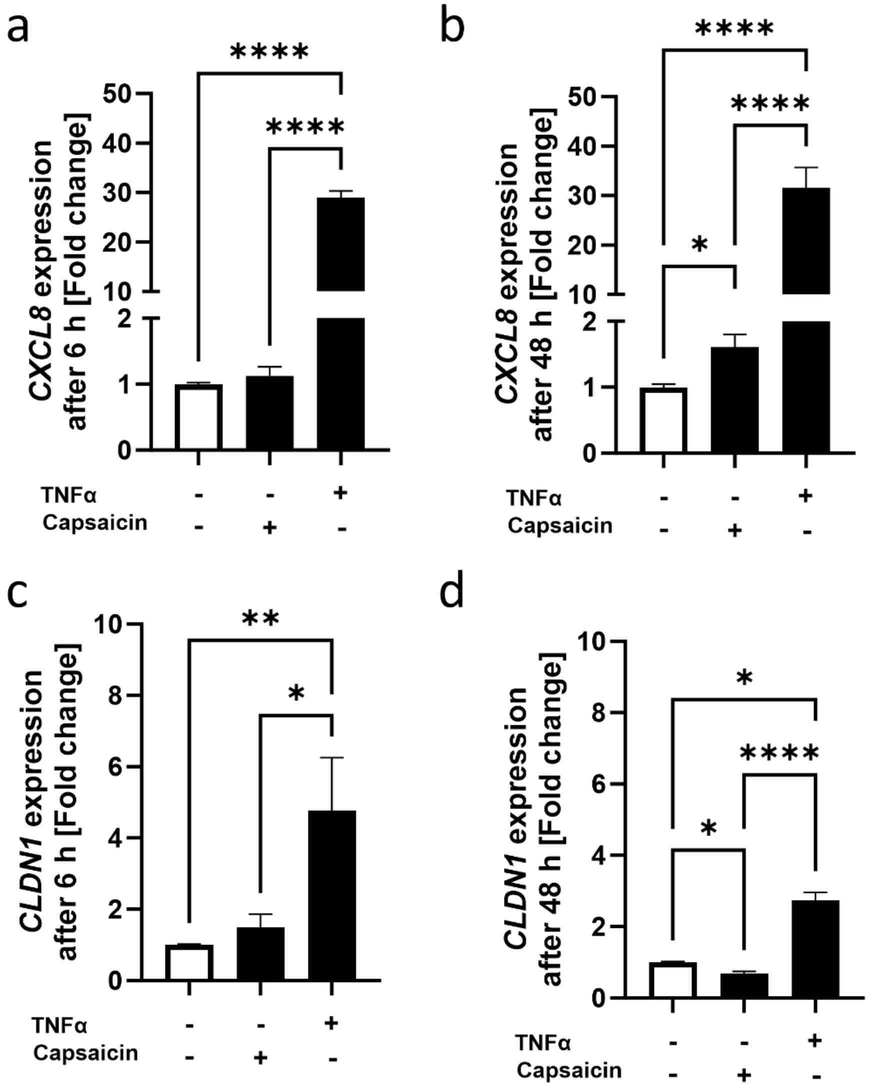

FIGURE S6. TNFα stimulation provokes an increase in *CXCL8* and *CLDN1* gene expression in HaCaT keratinocytes that exceeds changes elicited by capsaicin during pre-incubation. HaCaT keratinocytes were cultured in medium containing 0.06 mM Ca^{2+} for 4 days, pretreated with or without 10 µM capsaicin for 24 h and then treated with or without 20 ng/mL TNFα for 6 or 48 h. (a, b) TNFα stimulation provokes a significant increase in *CXCL8* gene expression. Changes caused by pre-incubation with capsaicin are negligible in comparison. (c) *CLDN1* gene expression is not significantly increased after 6 h by the capsaicin pre-treatment if no TNFα is used for stimulation. TNFα, conversely, increases the expression of *CLDN1* significantly. (d) At the 48 h timepoint after the pre-treatment with capsaicin there is a decrease in *CLDN1* gene expression when compared to the control. *CLDN1* expression is, in turn, upregulated when the cells are stimulated with TNFα after the pre-treatment. Data presented as fold change to the control (white bars). Relative gene expression of the different genes was measured with RT-qPCR and normalized to the geomean of the reference genes (i.e. GAPDH and HPRT1). (Statistics: mean + SEM; technical replicates: 3, biological replicates: 4, (a, b) Brown-Forsythe and Welch ANOVA with Dunnett’s T3 multiple comparisons

post hoc test, *$p<0.05$, ****$p< 0.0001$; (c-d) Kruskal Wallis test with Dunn’s multiple comparisons post hoc test *$p<0.05$, **$p<0.01$, ****$p<0.0001$).
